# Abdominal scar characteristics as a predictor of cervical stenosis after abdominal radical trachelectomy

**DOI:** 10.18632/oncotarget.9318

**Published:** 2016-05-12

**Authors:** Xiaoqi Li, Jin Li, Xingzhu Ju, Xiaojun Chen, Xiaohua Wu

**Affiliations:** ^1^ Department of Gynecologic Oncology, Fudan University Shanghai Cancer Center, Shanghai, PR China; ^2^ Department of Oncology, Shanghai Medical College, Fudan University, Shanghai, PR China

**Keywords:** abdominal radical trachelectomy (ART), cervical cancer, abdominal scar, Vancouver Scar Scale (VSS), cervical stenosis

## Abstract

To investigate whether abdominal scar characteristics could predict the occurrence of cervical stenosis after abdominal radical trachelectomy (ART), we conducted a retrospective study and investigated the relationship between abdominal scar characteristics and the occurrence of cervical stenosis in patients one year after undergoing ART. The abdominal scars were evaluated using the Vancouver Scar Scale (VSS). Seventy-two participants were enrolled in the study, including 15 (20.8%) women with cervical stenosis, and 57 (79.2%) without stenosis. Results showed that the mean abdominal scar score assessed by VSS was higher in patients with cervical stenosis (7, range: 1–10) compared to those without stenosis (4, range: 0–9) (*P* = 0.001). Incidence rates of cervical stenosis increased with the VSS score. For women with VSS scores of 0 to 4, 5, 6, 7, 8, 9 and 10, respectively, the occurrences of cervical stenosis were 6.1%, 16.7%, 16.7%, 27.3%, 37.5%, 50% and 100%. The cutoff point of VSS score was 7 according to the receiver operating characteristic (ROC) curve. Fourteen of the 15 stenosis happened either in patients without anti-stenosis tools (Foley catheters or tailed intrauterine devices) placed during the surgery or after the devices were removed. Our results demonstrated that VSS is an effective approach to assess the presence of cervical stenosis after ART. Women who have an abdominal scar with a VSS score > 7 have a high risk of developing isthmic stenosis without anti-stenosis tools in place.

## INTRODUCTION

With more unmarried and nulliparous women being diagnosed with early-stage cervical cancer, radical trachelectomy in combination with pelvic lymph node dissection has become an alternative to radical hysterectomy, helping those patients to maintain reproductive functions without sacrificing oncological safety [1–3]. However, since cervical stenosis is a major and unique postoperative complication, it can affect not only their quality of lives, but also patients' reproductive function. Stenosis of the cervix increases the risk of dyspareunia and might lead to anxiety [4]. Additionally, it can impede outflow of the menstrual volume, thereby causing menstrual problems or even amenorrhea [5]. Moreover, it is a potential cause of infertility and accounts for a large proportion of patients requiring assisted reproductive technologies [3, 6]. The incidence rates of cervical stenosis range from 0% to 73.3% with an average rate of 10.5% after RT; among patients with abdominal, vaginal, laparoscopic, and robotic radical trachelectomy, incidence rates are 11.0%, 8.1%, 9.3%, and 0%, respectively [7]. Therefore, predict the occurrence of cervical stenosis, especially after abdominal radical trachelectomy (ART), and take appropriate measures to prevent it seems crucial.

Cervical stenosis is caused by cervical canal adhesions and the contraction of scars after excision of the cervix. Because the formation of skin and cervical scars are both tissue-healing processes [8, 9], we speculate that there might exist correlations between the severity of abdominal scars and the presence of cervical stenosis. On the basis of the background described above, we conducted a retrospective study to investigate the relationship between abdominal scar characteristics and the occurrence of cervical stenosis for patients after ART. The association of skin scarring and cervical stenosis has not yet been investigated. Hope the present study can provide us a way to predict the appearance and minimize the risk of stenosis.

## RESULTS

Between June 2005 and December 2014, 152 patients with early stage cervical cancer underwent ART by the same group of surgeons at our institution. Eighty women were withdrawn: 9 patients had pathological types of botryoid sarcoma or adenosarcoma; 3 patients experienced recurrence; 2 received radiotherapy due to positive lymph nodes at final pathology; 37 did not have the anti-cervical stenosis tools removed; 9 had abdominal transverse incision and 20 did not followed. The rest of the 72 patients were included in the present study. Demographic and pathologic outcomes did not differ significantly between participants and non-participants.

Fifteen (20.8%) women were found to have cervical stenosis, and 57 (79.2%) had no stenosis. Among patients who had stenosis, 9 had severe stenosis and suffered from secondary amenorrhea due to total obliteration of the cervical canal; six patients with mild stenosis experienced prolonged menstruation or decreased volume but still maintained regular menstruation. Age, body mass index (BMI), marital status, parity before surgery, FIGO stage, tumor size, histology, prior LEEP or cone history, adjuvant chemotherapy, and follow-up times were shown in Table 1. The median follow-up was 33 (range: 12–126) months. There was no statistical difference in the demographic and pathologic outcomes between the women with and without cervical stenosis.

**Table 1:** Demographic and pathologic outcomes of the women with and without cervical stenosis

	Cervical stenosis (*N* = 15)	None cervical stenosis (*N* = 57)	*P*
Age at diagnosis (years), Mean (range)	33 (26–40)	31 (21–41)	0.119
Age at last follow-up, (years), Mean (range)	35 (28–42)	33 (23–45)	0.127
BMI, (kg/m2), Mean (range)	20.7 (17.6–26.0)	21.4 (16.6–33.9)	0.446
Marital status, *N*(%)			0.632
Married	14 (93.3%)	47 (82.5%)	
Unmarried	1 (6.7%)	10 (17.5%)	
Parity before the surgery, *N* (%)			0.093
0	6 (40.0%)	38 (66.7%)	
1	9 (60.0%)	17 (29.8%)	
2	0	2 (3.5%)	
FIGO stage, *N*(%)			0.052
IA1	5 (33.3%)	5 (8.7%)	
IA2	1 (6.7%)	4 (7.0%)	
IB1	9 (60.0%)	48 (84.3%)	
Tumor size (cm), Mean (range)	1.2 (0.2–3.0)	1.7 (0.1–3.5)	0.060
Histology, *N* (%)			0.258
Squamous	13 (86.7%)	49 (86.0%)	
Adenocarcinoma	2 (13.3%)	7 (12.3%)	
Adenosquamous	0	1 (1.7%)	
Prior LEEP or cone, *N* (%)	10 (66.7%)	25 (43.9%)	0.151
Adjuvant Chemotherapy, *N* (%)	1 (6.7%)	14 (24.6%)	0.169
Follow-up time (months), Median (range)	40 (12–107)	33 (13–126)	0.798

The mean scar height was greater for women with cervical stenosis (1.1, range: 0–4.0 mm) compared to women without stenosis (0.3, range: 0–2.0 mm) (*P* = 0.025). The median scar width was also longer in women with isthmic stenosis (9.7, range: 2.0–27.0 mm) versus women without stenosis (4.8, range: 0–12.0 mm) (*P* = 0.013). Table 2 shows the association of skin scar characteristics assessed by the Vancouver Scar Scale in the absence and presence of cervical stenosis. Women with cervical stenosis had a greater mean skin scar score (7, range: 1–10) as opposed to women without stenosis (4, range: 0–9) (*P* = 0.001). Specifically, women who had presented with a higher (*P* = 0.007) and more vascular (*P* = 0.006), pigmented (*P* = 0.023), and palpable scar (*P* = 0.007) were more likely to develop cervical stenosis after ART. Finally, two patients with VSS scores of 10 had abdominal keloids, and these two patients all developed severe stenosis.

**Table 2:** Abdominal scar characteristics evaluated using the Vancouver Scar Scale (VSS) in women with and without adhesions

Characteristic	Cervical stenosis (*N* = 15)	None stenosis (*N* = 57)	*P*
Vascularity, *N*(%)			0.006
0 Normal	2 (13.3%)	29 (50.9%)	
1 Pink	3 (20.0%)	7 (12.3%)	
2 Red	4 (26.7%)	16 (28.1%)	
3 Purple	6 (40.0%)	5 (8.7%)	
Pigmentation, *N*(%)			0.023
0 Normal	0 (0%)	8 (14.0%)	
1 Hypopigmented	2 (13.3%)	10 (17.5%)	
2 Mixed	2 (13.3%)	21 (36.8%)	
3 Hyperpigmented	11 (73.4%)	18 (31.7%)	
Height, *N* (%)			0.007
0 Flat	3 (20.0%)	29 (50.9%)	
1 < 2 mm	9 (60.0%)	24 (42.1%)	
2 2–5 mm	3 (20.0%)	4 (7.0%)	
3 > 5 mm	0 (0%)	0 (0%)	
Pliability, *N*(%)			0.007
0 Normal	2 (13.3%)	29 (50.9%)	
1 Supple	4 (26.7%)	27 (47.4%)	
2 Yielding	9 (60.0%)	1 (1.7%)	
3 Firm	0 (0%)	0 (0%)	
4 Ropes	0 (0%)	0 (0%)	
5 Contracture	0 (0%)	0 (0%)	
Total score, Mean (range)	7 (1–10)	4 (0–9)	0.001

Incidence rates of cervical stenosis increased with the VSS score. Among women with a VSS score of 0 to 4, only 2 women developed isthmic stenosis (6.1%). In women who had a VSS of 5 to 10, the incidence of cervical stenosis was 16.7% (1/6), 16.7% (1/6), 27.3% (3/11), 37.5% (3/8), 50% (3/6), and 100% (2/2), respectively.

The ROC-AUC for the prediction of cervical stenosis by VSS score was 0.78 (95% confidence interval of 0.63–0.92). According to the ROC analysis, a threshold value of a VSS score of 7 for the prediction of cervical stenosis was calculated (sensitivity 73.3%; specificity 71.9%; Youden Index 0.45).

To prevent cervical stenosis, we usually inserted a Foley catheter or a tailed intrauterine device (IUD) in the uterine cavity during the procedure. Among 15 patients who had isthmic stenosis, 3 did not have an anti-stenosis device inserted, 2 had a Foley catheter and 10 had a tailed IUD placed. Details of the placement of the tools and their relationship with cervical stenosis as well as VSS score in the 15 patients were shown in Table 3. Fourteen of the 15 patients developed cervical stenosis when the devices were not in the in the uterine cavity (did not inserted during the surgery, removed or the tail of the IUD dropped). One patient had stenosis and intrauterine adhesions even with the IUD in place. All had cervical dilation and 14 succeeded. One woman with an abdominal keloid developed severe cervical stenosis and intrauterine adhesions soon after the IUD was removed and did not resume menstruation even after dilation and adhesiolysis. Eight women had dilation more than once. However, in the 57 patients without stenosis, 7 did not have an anti-stenosis device inserted, 6 had a Foley catheter and 44 had a tailed IUD placed. The mean duration time was 30 (4–60) days and 8.6 (0.5–34) months, respectively for patients with Foley catheters and tailed IUDs placed. None developed stenosis after the device was removed.

**Table 3:** Details of the anti-stenosis tools, cervical stenosis and the Vancouver Scar Scale (VSS) score in patients with cervical stenosis

No.	Anti-stenosis tools		Cervical stenosis		VSS score
	Tools	Duration time	Grade	Occurrence time	
1	IUD	14 months	Severe	Within one month after the tail of the IUD dropped	10
2	IUD	12 months	Severe	Within one month after removal of the IUD	10
3	IUD	1 week	Severe	Within one month after surgery	9
4	None	/	Severe	Within one month after surgery	9
5	IUD	6 months	Severe	Within one month after removal of the IUD	8
6	IUD	6 months	Severe	Within one month after removal of the IUD	8
7	IUD	17 months	Severe	Within one month after removal of the IUD	7
8	IUD	9 months	Severe	Within one month after removal of the IUD	6
9	IUD	3 months	Severe	Within one month after surgery	1
10	Catheter	10 days	Mild	One year after surgery	9
11	Catheter	1 month	Mild	One year after surgery	8
12	IUD	3 months	Mild	Within one month after removal of the IUD	7
13	None	/	Mild	Half a year after surgery	7
14	None	/	Mild	Half a year after surgery	5
15	IUD	1 month	Mild	Half a year after surgery	1

IUD: intrauterine device; Severe: severe stenosis, completely obstructed of the cervical os; Mild, mild stenosis: stenosis but not atresia.

## DISCUSSION

Cervical stenosis is a major postoperative complication and is a main cause of infertility following radical trachelectomy [6, 10]. However, so far there have been no studies that specifically investigated the incidence of cervical stenosis, and no definitive guideline exists for the prediction and prevention of such a complication. This study showed that the abdominal scar characteristics of women were associated with the presence of cervical stenosis after ART. This new finding could be used as a simple clinical tool to estimate the occurrence of cervical stenosis after ART, and may enable clinicians to prevent and minimize the risk of stenosis.

In our study, women who had cervical stenosis had a higher mean VSS score than those without stenosis. The presence of cervical stenosis increased with an increased VSS score. If the VSS score was between 0 and 4 one year after ART, only 6.1% of the patients developed isthmic stenosis. However, if the VSS score was 10, 100% of the patients developed stenosis. The cutoff point was 7 according to the AUC analysis.

Anti-stenosis tools, especially the tailed IUD, could effectively prevent the occurrence of cervical stenosis, particularly for those with a high VSS score after ART. According to our study, for patients suffered from cervical stenosis, almost all of the stenosis happened when patients did not have anti-stenosis tools placed in the uterine cavity (without anti-stenosis tools placed during the surgery or after the tools was removed). Ten of the 15 cases happened less than one month after surgery or after the tools were removed. Because patients with a VSS score of abdominal scar > 7 were more likely to develop cervical stenosis after ART and the tailed IUD could be kept in place much longer than the Foley catheter, we highly recommend a routinely utilize a tailed IUD during the surgery. This could decrease the occurrence of stenosis and minimize the infertility caused by complete obstruction of the cervical os. For patients who had an abdominal scar with a VSS score > 7 one year after ART, the IUD should be placed until the patients plan to conceive. For patients with a VSS score ≥ 10, assisted-reproduction technologies are highly recommended, and the IUD should be placed until the transfer process.

The Vancouver Scar Scale is the first validated burn scar assessment scale, published by Sullivan et al.[11] in 1990, and remains the most widely used scale to access post-burn and post-surgical scars within a clinical setting [12, 13]. The higher the sum of the scores, the more marked or abnormal the scarring [12]. Thompson et al. used a VSS score of > 7 as a way of identifying hypertrophic scars [14]. In this study, we discovered a relationship between the VSS score and the presence of cervical stenosis after ART. Additionally, we found that patients with a VSS score > 7 as a cutoff point determined the occurrence of stenosis. This finding shows that the VSS is an effective tool in the prediction of cervical stenosis after ART.

The shared characteristics between abdominal scarring and cervical stenosis were due to the following reasons: first, they are all results of the wound-healing process. Therefore, they both have many common cellular and molecular mechanisms, and both involve the process of inflammation, new tissue formation, and remodelling [9, 15–17]. Second, they were all influenced by wound degree, infection, hormone level, and the individual's ethnic background. It is reported that deep wounds [18–20], infection [21–23], young age [24–26], and non-White people [27, 28] can increase the chance of pathological scar formation both in the skin and in the cervix. Third, pathological skin scars especially keloids have a substantial evidence for family history and genetic susceptibility. Patients with abdominal pathological scars are more likely to develop severe scars compared to those without such characteristics [15, 29]. In our study, two patients with abdominal keloids and a VSS score of 10 developed severe stenosis after ART, and one never resumed menstruation due to secondary intrauterine organization. Therefore, patients with keloids should be highly attuned to the presence of cervical stenosis after ART.

To the best of our knowledge, this is the first study of its kind that has confirmed an association between skin scar characteristics and the presence of cervical stenosis after ART. This finding provides a way to recognize patients with a high risk of cervical stenosis after ART, enabling surgeons to take precautionary measures to prevent it. All the surgeries were performed by the same group of surgeons with the same instruments, and the techniques used in each surgery were accurately standardized. Additionally, because scarring generally stops hypertrophy after 12 months of surgery, we enrolled patients with a follow-up time of more than 12 months in order to decrease the effect of scar generation on the accuracy of the study [30, 31]. However, our study is still limited due to the small number of enrolled patients and its retrospective nature. Further prospective clinical studies with more patients are required.

## MATERIALS AND METHODS

### Study design

We designed a retrospective study to examine the relationship between abdominal scar characteristics and the incidence of cervical stenosis after ART. The appearance of patients' abdominal scars were recorded during follow-up from July 2015 to December 2015 and the conditions of the new cervix after surgery were acquired from a prospectively recorded database. Eligible patients were those who: (1) met the inclusion criteria for and underwent ART from June 2005 to December 2014 by the same group of surgeons at our institution; (2) had a follow-up time of greater than or equal to 12 months after ART; (3) experienced only one surgery at the same incision and did not suffer from wound infection; (4) did not have anti-stenosis tools inserted, such as tailed intrauterine devices (IUDs) or Foley catheters during ART, or had the tools removed more than 3 months at the follow-up; and (5) had been disease-free without recurrence during follow-up. Ethics approval was granted by the institutional review board in our hospital.

### Scar assessment

All scars were assessed independently by two observers on the same day when the patients were lying in a supine position with the scar exposed in bright light. If the data varied, another researcher was required to assess the scar at the same day and the results were recorded according to the one with the highest frequency. To avoid bias, there were only three experienced observers and the observers who assigned the scar characteristics were not notified of the cervical condition of the patients.

All scars were assessed for height, width, and four scar parameters using the Vancouver Scar Scale (VSS), which included vascularity (normal, pink, red, or purple), pigmentation (normal, hypopigmented, mixed, or hyperpigmented), height (flat, < 2 mm, 2–5 mm, or > 5 mm), and pliability (normal, supple, yielding, firm, ropes, or contracture) [12]. Each variable contained ranked subscales that could be summed to obtain a total score ranging from 0 to 14, with 0 representing normal skin and a higher score representing a more marked or abnormal scar.

### Cervical appearance

Cervical stenosis was diagnosed if patients had ever presented with stenosis of the cervix. Data of patients' cervical appearance and demographic and pathologic outcomes were collected retrospectively from a prospectively maintained database. Cervical stenosis was defined as the condition that lead to prolonged duration of menses by more than 10 days, obviously decreased menstrual volume by greater than 2/3, or amenorrhea due to cervical reasons (after excluding endometrial, ovarian, and hypothalamus–pituitary factors, among others). Change in menstrual blood volume was estimated according to the number and saturation degree of the used menstrual pads. Stenosis was graded as mild (stenosis but not atresia) or severe (completely obstructed).

### Technique

ART procedures were performed by the same group of surgeons at our institution. Details of the techniques were published previously [2]. A Foley catheter or a tailed IUD was placed in the uterine cavity to prevent cervical stenosis during the procedure at some of the patients. The Foley catheter was kept for several weeks. This tailed IUD was removed 3–6 months after surgery at an early stage of our study. Since April 2011, we preferred to keep the device in place until patients attempted to conceive.

A long midline incision extending from the pubic symphysis to 2 cm above the umbilicus was conducted during the surgery. The parietal peritoneums and fascia were closed with Polysorb 1 sutures (Covidien, Shanghai, China), while the subcutaneous fat was closed with interrupted sutures using Polysorb 3–0 threads (Covidien, Shanghai, China). The skin was sutured continuously with Polysorb 4–0 (Covidien, Shanghai, China).

### Statistical analysis

Statistical analysis was performed using SPSS 21.0 software (Chicago, IL, USA). Categorical variables were described with proportions and continuous variables with mean/median and range. The association between the presence of cervical stenosis and other categorical variables was examined using the chi-square test. Continuous variables were analyzed by the *t*-test. Odds ratio estimates were calculated with 95% confidence intervals. The VSS score to predict the occurrence of cervical stenosis was assessed by calculating the receiver operating characteristic area under the curve (ROC-AUC). Threshold values were determined by considering values that yielded the greatest sensitivity and specificity by calculating the Youden Index. *P* values less than 0.05 were considered statistically significant.
